# Frequencies of Gag-restricted T-cell escape “footprints” differ across HIV-1 clades A1 and D chronically infected Ugandans irrespective of host HLA B alleles^☆^

**DOI:** 10.1016/j.vaccine.2015.02.037

**Published:** 2015-03-30

**Authors:** Jennifer Serwanga, Ritah Nakiboneka, Susan Mugaba, Brian Magambo, Nicaise Ndembi, Frances Gotch, Pontiano Kaleebu

**Affiliations:** a MRC/UVRI Uganda Research Unit on AIDS, Entebbe, Uganda; b Department of Immunology, Imperial College, Chelsea & Westminster Hospital, London, United Kingdom; c London School of Hygiene and Tropical Medicine, United Kingdom

**Keywords:** HIV-1, Gag, Clades A1 and D, HLA B alleles, Escape mutations, Selection pressure

## Abstract

**Objective(s):**

We evaluated relationships between critical Gag T-cell escape mutations and concomitant T-cell responses to determine whether HLA-restricted Gag mutations that confer protection, occur at similar rates in a population infected with mixed HIV-1 clades A1 and D viruses.

**Methods:**

Assessment of Gag selective pressure, and adaptive T-cell functions to KAFSPEVIPMF (KF11), ISPRTLNAW (ISW9) and TSTLQEQIGW (TW10) Gag epitopes were combined with host HLA to assess correlations with rates of critical epitope escape mutations in clades A1- (*n* = 23) and D- (*n* = 21) infected, untreated subjects. Infecting clades and selection pressure were determined from the gag sequences.

**Results:**

Overall, Gag escape mutations A163X in KF11 were detected in 61% (14/23) A1- infected compared to 5% (1/21) in D-infected subjects (*p* = 0.00015). Gag mutations I147X in the ISW9 epitope were seen in 43%: (10/23) clade A compared to 5%: (1/21) clade D infected subjects, *p* = 0.007, Fisher's Exact test. Both mutations were more frequent in clade A1 infection. Frequencies of the measured epitope-specific T-cell responses were comparable across clades. Peptide binding affinities for the restricting HLA alleles did not differ across clades. Overall, selection pressure on the Gag protein was significantly greater in clade A than in clade D sequences.

**Conclusions:**

These findings imply that HIV-1 vaccine strategies designed to target structurally constrained T-cell epitopes may be further challenged by clade-driven outcomes in specific HLA-restricted Gag epitopes. Equally, the data are line with slower HIV-1 disease progression in clade A infection; and raise hope that increased selective pressure on Gag may be protective irrespective of host HLA alleles.

## Highlights

• A and D infected subjects even though they bear the same presenting HLA alleles, and live in the same environment. Escape mutations that are known to confer survival advantage were more frequent in clade A-infected subjects irrespective of host HLA alleles.
• There was no evidence to link this difference in outcome to the evaluated adaptive T-Cell responses (IFN-γ responses and polyfunctional responses) to those key structurally constrained Gag epitopes.
• However, we have demonstrated that there was significantly greater selective pressure on the Gag protein of clade A than that of clade D.
• The data are in line with the known faster disease progression in clade D than clade A infected individuals.
• The data also highlight that the current difficulties in formulating a global HIV vaccine design will be further challenged by clade associated differences in outcome.

## 1 Introduction

HIV-1 has evolved into distinct clades that influence disease outcome differently; for example, infection with HIV-1 clade D results in faster disease progression than infection with clade A [1–3]. Contrasting outcomes are partly linked to differences in the quality of induced virus-specific T-cell responses [4–6]. Protectiveness of the induced T-cell responses is partly linked to the superior secretion of HIV-specific Perforin, polyfunctionality of the responses [5,7–9] and greater targeting of Gag [5,10,11]. The Gag p24 region is highly immunogenic, but structurally constrained. Immune pressure on Gag p24 epitopes yields critical epitope escape mutations [11–13] that impair virus replication, affording survival advantage to infected hosts [5,10,14] and vaccine recipients [15]. Host HLA B alleles exert the greatest influence on HIV-1 disease outcome; HLA B*57 and B*5801 alleles are associated with slower disease progression [16–18]. Of the HLA alleles studied in Caucasian, African, Asian, and Hispanic populations to date, HLA B*57 imparts the greatest impact on virological control [19]; this outcome is largely achieved through T-cell targeting of Gag TSTLQEQIAW (‘TW10′, Gag 240–249), KAFSPEVIPMF (‘KF11′, Gag 162–172) epitopes [20].

Gag-associated protection is partly achieved through targeting the conserved and highly constrained KAFSPEVIPMF (KF11), ISPRTLNAW (ISW9) and TSTLQEQIGW (TW10) [11,12,21] Gag p24 epitopes; yielding critical escape mutations that reduce virus replication [11–13,21]. The TW10 response dominates in acute HIV infection of HLA B*57 and B*5801 subjects yielding T242N mutations that are associated with lower viral loads over time [22,23]. Reversion of the transmitted T242N to wild-type sequence implies that this mutation affects virus fitness [24]. The KF11 epitope sequence is identical in both clades A and D consensus sequences. In subjects with presenting alleles, chronic infection is dominated by the HLA-B*57-restricted KF11 response and consequent A146X escape mutations that impair virus replicative ability [12,13,24]. Effects of the A163X are partially compensated for by a subsequent S165N substitution [25].

HLA-restricted imprints in structurally compromised epitopes would be expected to follow predicted patterns in subjects with the same presenting alleles; however, this has not always been the case [26,27]. It is not clear how concurrent T-cell responses are attributable to this outcome. Here, we combined adaptive T-cell responses, host HLA alleles and the KF11, ISW9 and TW10 epitope sequences to evaluate how frequencies of critically relevant epitope escape correlate with concurrent T-cell responses across clades A and D infection among subjects living in the same environment.

## 2 Methods

### 2.1 Study population and evaluation of immune responses

HIV-1 infected, therapy-naïve subjects were recruited for a cross sectional evaluation. Participant plasma viral loads (HIV RNA copies per ml), CD4+ T-cell counts (cells/μl) and HLA alleles were determined as previously described [10]. Infecting clades, estimation of selection pressure and KF11, ISW9 and TW10 epitope diversities were determined from the *gag* sequences. Cryopreserved peripheral blood mononuclear cells (PBMC) from 44 subjects were initially evaluated for IFN-γ response to KF11, ISW9 and TW10, Table 1. Sixteen subjects were further assessed for simultaneous secretion of IFN-γ, IL-2, TNF-α and Perforin in response to KF11, ISW9 and TW10, using intracellular cytokine staining assay. Selection for flowcytometry evaluations was based on cell availability, presence of A163G mutations in the KF11epitope and/or possession of HLA B*57 or B*5801 alleles. Uganda Virus Research Institute Ethics Review Board and the Uganda National Council of Science and Technology reviewed and approved this study. All subjects provided written informed consent for collection and subsequent evaluation of their specimens.

### 2.2 Estimation of synonymous (dS) and non-synonymous (dN) rates

Selective pressure was computed from the rates of non-synonymous (dN) and synonymous (dS) substitutions. The contributions of dN and dS rates to the overall substitution rate were estimated based on the posterior substitution rates estimated by Bayesian analysis. Their estimation was performed using a local codon model, as implemented in HYPHY [28]. We used the MG94xHKY85 codon model [29], an extension of the classical MG94 model with estimation of equilibrium codon frequencies, using nucleotide frequencies specific to each codon position.

### 2.3 HIV-1 Gag peptides

Peptides used in the Interferon (IFN)-γ ELISpot assays were 15 amino acids (a.a) long and overlapped by 10 a.a *(*https://www.aidsreagent.org/Index.cfm). They encompassed HIV-1 Gag HXB2 residues: (a) 162–172 (KAFSPEVIPMF, designated KF11; (b) 147–155 (ISPRTLNAW, designated ISW9); and (c) 240–249 (TSTLQEQIGW, designated TW10). United Peptide Biosystems, Inc designed the flowcytometry peptides that represented circulating variants of KF11, ISW9 and TW10. These peptides comprised wild type KAFSPEVIPMF (KF11-1) epitope and three variants KGFSPEVIPMF (KF11-2); KAFNPEVIPMF (KF11-3); and RGFSPEVIPMF (KF11-4)]; wild type ISPRTLNAW epitope designated (ISW9-1) and one variant ASPRTLNAW designated (ISW9-2)]; and wild type TSTLQEQIGW epitope (TW10-1) and one variant TSNLQEQIGW designated (TW10-2)]. Individual peptides were used at a final concentration of 1 μg/ml in all PBMC stimulations.

### 2.4 The ELISpot assay

Virus-specific IFN-γ responses were quantified using ELISpot assay, as previously described [30]. Responses were enumerated as spot forming units per million PBMCs (SFU/10^6^ PBMCs). The test acceptance criteria were: ≥300 SFU per PHA well; ≤100 cumulative spots in all the six background wells; and ≤5 cumulative SFU in the two wells that contained media only. Test wells with ≥100 net SFU/10^6^ PBMCs after subtracting three times the background were considered positive.

### 2.5 Functional avidity of the KF11 peptides

The IFN-γ ELISpot assay was used to evaluate five HLA B57 subjects for binding affinities to the 11-mer KF11 peptide; the study subjects were selected based on PBMC availability. Binding affinities were determined using duplicate 2-fold serial peptide dilutions ranging from 2 μg/ml to 4 pg/ml), as described elsewhere [31]. Peptide concentrations that yielded half the maximum number of sport forming units were determined from a sigmoidal dose-response curve fit using Graph Pad Prism 5. Functional avidities were expressed as half-maximal stimulatory peptide doses (SD 50%).

### 2.6 Fluorochrome antibodies

Dead cells, B-cells, and monocytes were excluded using Aqua (L34957, Invitrogen), CD19 APC Alexafluor750 (1072337A, Invitrogen) and CD14 APC Alexafluor750 (773927B, Invitrogen) antibodies, respectively. T-cells were defined using CD3 (brilliant violet 570, B152103, Biolegend), CD8 (pacific blue, 22416, BD Bioscience) and CD4 (PE-Cy5.5, 1049514A, ebiosciences) surface staining antibodies. We defined CD4+ T-cells as the CD3+CD8−CD4+ T-cell phenotype, and CD8+ T-cells as the CD3+CD4−CD8+ T-cell phenotype. Virus-specific CD4+ and CD8+ T-cell responses were determined using intracellular staining for IFN-γ (Alexafluor 700, 21128, BD Biosciences); IL-2 (APC, 341116, BD Biosciences); Perforin (FITC B-D48 clone, F111124, Diaclone); and TNF-α (PE-Cy7, E07677-1632, ebiosciences). Simultaneous secretion of IFN-γ, IL2, Perforin and TNF-α in response to stimulation with the respective HIV-1 peptides was measured. We defined polyfunctionality as the concurrent secretion of three or more T-cell functions.

### 2.7 Intracellular cytokine staining assay

**Supplementary Fig. 1:**
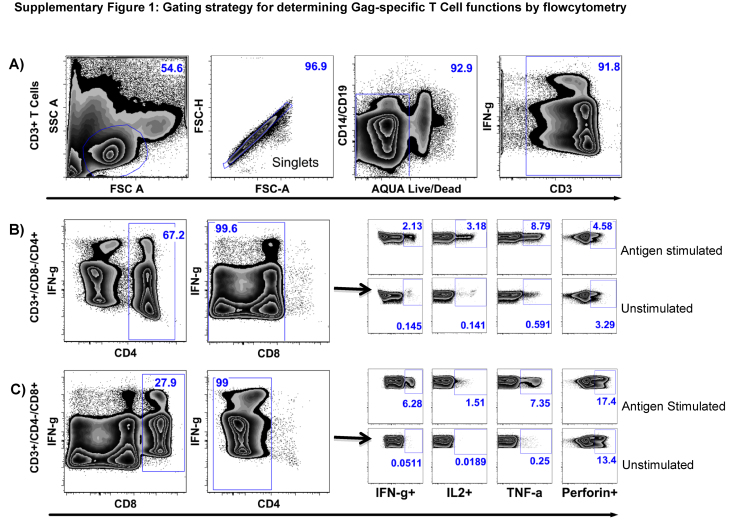
Gating strategy for detection of Gag induced T-cell functions. Intracellular cytokine-staining (ICS) assay was used to assess simultaneous detection of IFN-γ, IL-2, TNF-γ and Perforin responses. (A) Shows sequential gating to identify CD3+ T-cell populations. The first gate selects lymphocytes; the second gate eliminates doublets; the third gate eliminates B cells, monocytes and dead cells; and the fourth gate restricts subsequent analyses to live, bright and dim CD3+ T-cells. Within the CD3+ T-cells, CD4+CD8+ double positive T-cells were excluded to limit ensuing analyses to single positive CD4+ T-cells, (B) and CD8+ T-cells (C). Analyses of T-Cell responses to unstimulated and antigen-stimulated specimens are illustrated.

HIV-specific T-cell polyfunctional responses were assessed using intracellular cytokine staining assay, as previously described [32], but with slight modifications. Briefly, thawed and rested PBMCs were incubated with 1 μg/ml peptide for 6 h at 37 °C in a 5% CO_2_-in air, humidified environment, in the presence of Golgi Plug™ and CD28/CD49d co-stimulatory antibodies. Negative controls were PBMCs incubated as above but without peptides. Positive controls were PBMCs incubated with *Staphyloccoccus* enterotoxin B (SEB) instead of peptides. Stimulated PBMCs were then washed in 10% FBS in PBS, and incubated with Aqua viability dye before surface staining for T-cell lineage markers. The PBMCs were subsequently intracellularly stained, for the simultaneous expression of Interferon (IFN)-γ IL and Perforin. Flowcytometry data was analyzed using FlowJo (version 9.5.3, TreeStar), Pestle (version 1.6.2) and SPICE (Version 5.3101) [33] software. The gating strategy for definition of IFN-γ, IL-2, TNF α and Perforin is illustrated in Supplementary Fig. 1.

Supplementary material related to this article can be found, in the online version, at http://dx.doi.org/10.1016/j.vaccine.2015.02.037.

### 2.8 Statistical analysis

Statistical analyses were performed using Stata V 10.0 software (Stata Corp, TX, USA) and Graph Pad 5.0 (GraphPad Software, Inc., San Diego, CA, USA). Graphical presentations were performed using SPICE and Graph Pad software. Continuous data is presented as medians, with their interquartile ranges (IQR). Medians were evaluated using Mann Whitney test if there were two groups; or Kruskal–Wallis Rank Sum test if there were more than two groups. Proportions were compared using the Fisher's Exact test. Means were compared using the Student's *t*-test. *p* values ≥0.05 were considered significant.

## 3 Results

### 3.1 Cohort characteristics and infecting HIV-1 clades

The study evaluated 14 males (28%) and 36 females (72%). Their median age was 37 years (IQR 31–41), median CD4+ T-cell counts were 538 cells/μl (IQR 484–647) and median plasma viral loads were 15,550 RNA copies/ml (IQR 3450–52,400). Infecting HIV-1 clades based on the *gag* gene were 46% A1 (23/50), 42% D (21/50), 6% CRF10_CD (3/50), 2% A1/C (1/50), 2% C (1/50) and 2% unclassifiable (1/50); GenBank accession numbers HQ702686-HQ702731 [10]. Plasma viral loads and CD4+ T-cell counts were relatively similar across clade A1-infected subjects (12700; 2280–73600 RNA copies/ml) and 510; IQR 444–578 CD4+ T-cells/μl); and clade D infected subjects (13100; 6070–162000 RNA copies/ml) and 579; IQR 487–650 CD4+ T-cells/μl, respectively. The WHO clinical classifications of HIV-1 infection were 24% (12/50) stage I, 64% (32/50) stage II and 12% (6/50) stage III. All subsequent analyses are based on 44 subjects that were infected with clades A1 (*n* = 23) and D (*n* = 21), Table 1.

### 3.2 The KF11 epitope escape A → X at Gag position 163 (A163X) was associated with clade A1 infected subjects irrespective of host alleles

**Fig. 1:**
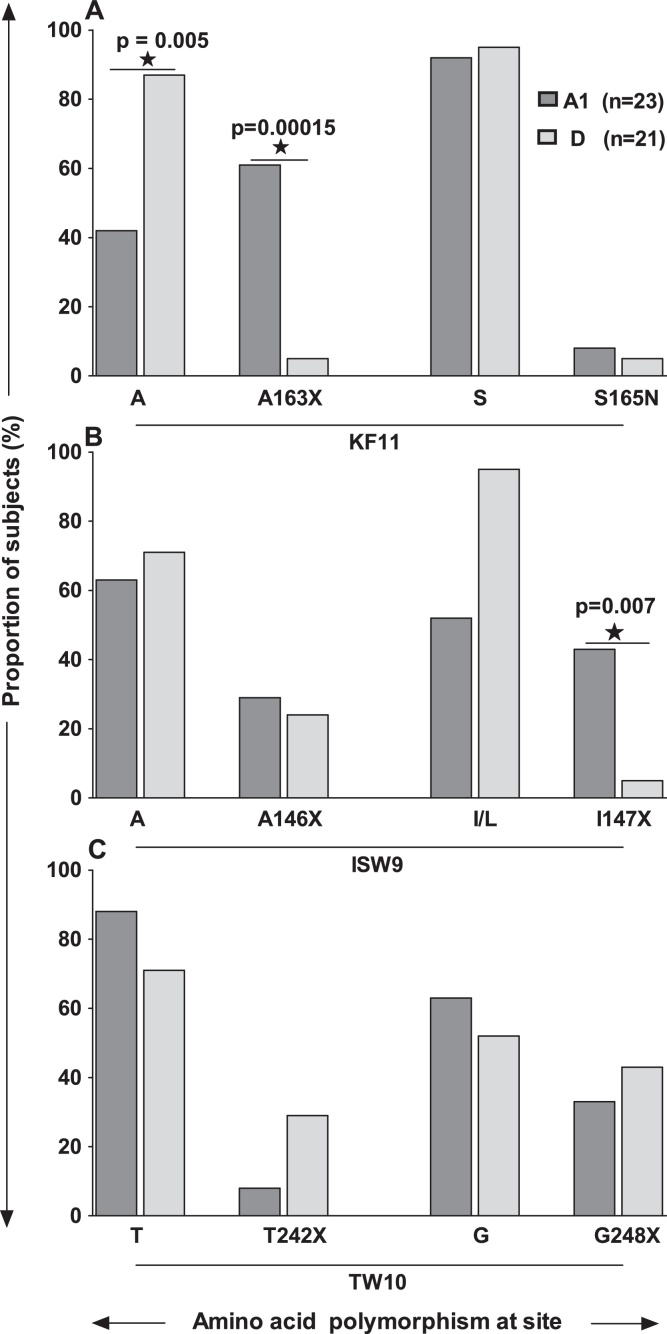
Frequencies of KF11, ISW9 and TW10 epitope variants across clades. This figure illustrates frequencies amino acids variation at Gag residues 163 and 165; Gag residues 146 and 147; and Gag residues 242 and 243. Frequencies of the A163X mutation (where X = G, V or S at residue 163), (A) I147X mutation (where X = L, V, F, W or M at residue 147), (B) and the T242X mutation (where X = N or S), (C), are compared in clades A1 (*n* = 23) and D (*n* = 21) subjects. Compensatory mutations linked to the above escape mutations are also illustrated.

The KF11 amino acid sequences in this population were identical to the corresponding HXB2 epitope sequences in both clades A1- and D-infected subjects. Epitope polymorphisms occurred at the Serine residue 165 (S165N); and at Alanine residue 163 (A163X) where Alanine was substituted with Glycine (G: *n* = 13), Valine (V: *n* = 1) or Serine (S: *n* = 1). The presence of A163X was associated with clade A1 infected subjects. Overall, A163X was found in 15 of 44 subjects (34%); comprising 61% (14/23) clade A1 and 5% (1/21) clade D; *p* = 0.00015, Fisher's Exact test, Fig. 1A. Of the 8 HLA B*57/5801 subjects, A163X occurred in 80% clade A1 (4/5) compared to 0% in clade D (0/3); *p* = 0.03, Table 1. Compensatory mutation S165N is known to partially restore A163X-associated impairment of virus replication [25]. Overall, S165N mutation was less frequent (5%, 2/44). Of 2 subjects with S165N, the clade D infected subject had higher plasma viral loads (UG017; 178,000 RNA copies/ml) than the clade A subject (UG065; 4240 RNA copies/ml).

### 3.3 I147X compensatory mutation in the ISW9 epitope was more frequent in clade A1-infected patients

ISW9 epitope polymorphisms comprised the A146X escape in the epitope flanking region (X = G [2], N [3], P [6], S [1] or V [1]); and the I147X compensatory mutations (where X = V [8], F [1], W [1] or M), that are known to restore immune recognition of ISW9 (29). The presence of Leucine (L) instead of Isoleucine (I) at Gag residue 147 was consensus in sequences from both the clades A- and D-infected subjects. Therefore, I147L is not considered to be a polymorphism in our I147X analyses. Overall, I147X was found in 25% (11/44) subjects, comprising 43% (10/23) clade A1 infected, and 5% (1/21) clade D infected, irrespective of host HLA alleles; *p* = 0.007; Fisher's Exact test, Fig. 1B. Polymorphism A146X was found in 13/44 (30%) subjects, and occurred at comparable frequencies across clades A1- (30%, 7/23) and D (29%, 6/21), Table 1.

### 3.4 Mutations in the TW10 epitope occurred at comparable frequencies across clades

Polymorphisms in the TW10 epitope included escape mutation T242X in 16% of the subjects (where X = N [*n* = 4] or S [*n* = 3]); and the compensatory mutation G248X seen in 39% of the subjects (where X = A [*n* = 15], Q [*n* = 1] or T [*n* = 1];), Table 1. There was no significant difference between frequencies of T242X ([4%; 1/23] vs. [29%; 6/21]) and G248X ([30%; 7/23] vs. [48%; 10/21]) across clades A1 and D, respectively, Fig. 1C.

### 3.5 KF11-, ISW9- and TW10-induced IFN-γ frequencies were comparable across clades

Of 44 subjects, 6 (14%), 10 (23%) and 1 (2%), had measurable IFN-γ responses to ISW9, KF11 and TW10, respectively (Table 1). The proportion of subjects targeting KF11 and ISW9 did not significantly differ across clade A1 (5/23 and 3/23) and clade D (5/21 and 3/21), respectively. In 10 subjects that targeted KF11, median IFN-γ magnitudes determined by ELISpot, were comparable across clade A1 (1875: IQR 820–2105 SFU/10^6^ PBMCs) and D (940: IQR 645–3810 SFU/10^6^ PBMCs), *p* = 0.75, Mann Whitney test. Similarly, in 6 responders to ISW9, median IFN-γ magnitudes did not significantly across clade A1 (190; IQR 125–515 SFU/10^6^ PBMCs) and clade D (130; IQR 110–3593 SFU/10^6^ PBMCs); *p* = 0.83, Mann Whitney test. Two of eight subjects with HLA B*57 and *5801 and A163X had detectable HIV-specific IFN-γ responses. Median plasma viral loads were significantly lower in the subjects with detectable IFN-γ responses (1814; IQR 488–3140 RNA copies per ml) compared to those without (14050; IQR 11600–27200 RNA copies per ml); *p* = 0.045, Kruskal–Wallis rank test.

### 3.6 In HLAB*57 subjects with the A163X mutation, virus-specific CD8+ T-cell polyfunctionality were at similar frequencies across clades

**Fig. 2:**
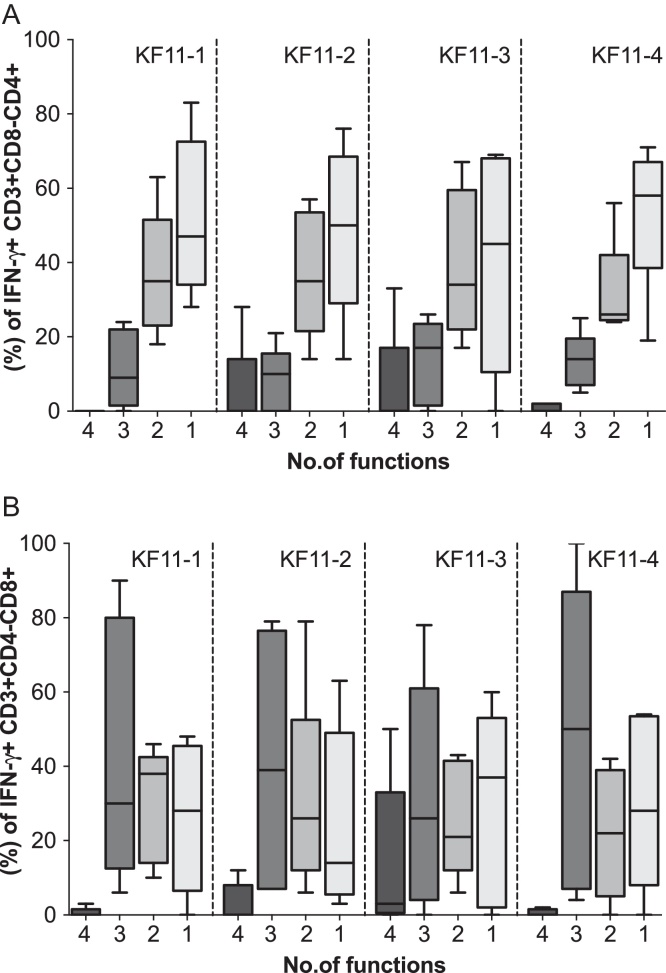
HIV-specific T-cell responses to KF11 epitope variants were detectable in subjects with A165X. This figure illustrates expression of HIV-specific IFN-γ, IL-2, TNF-α and Perforin responses to four KF11 epitope variants in allele-restricting subjects possessing the A165X mutation. Simultaneous detection of four, three, two and one HIV-specific functions (IFN-γ, Perforin, lL-2 and TNF-α) is evaluated in CD4+ and CD8+ T-cells of HLA B57*/5801 subjects (A and B).

**Fig. 3:**
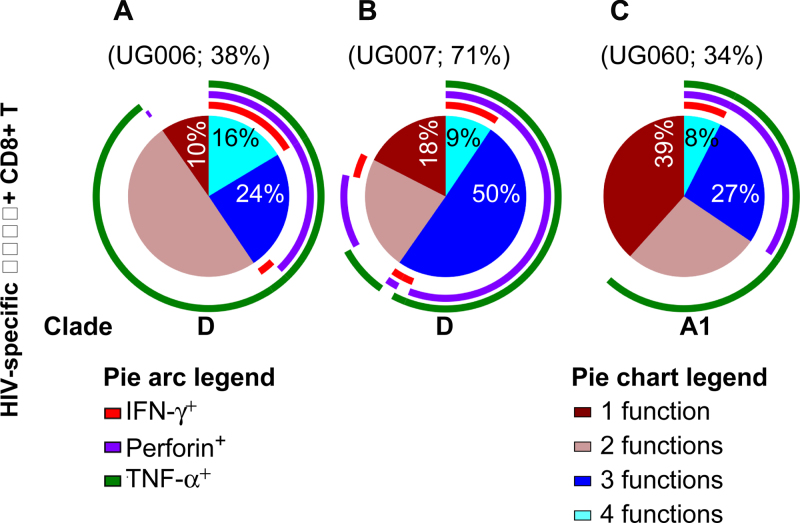
Virus-specific CD8+ T-cell polyfunctionality was not superior in A163X-bearing, clade A-infected HLAB*57 subjects. Subject PBMCs were stimulated with pooled KF11 variants, and assessed for the simultaneous expression of IL-2, IFN-γ, TNF-α and Perforin, Antigen-specific IL-2-secreting cells CD8+ T cells were delineated using Boolean gating. The contribution of Perforin, IFN-γ and TNF-α to the total response was then evaluated on the defined virus-specific IL-2 secreting T cells. Polyfunctionality was defined as the simultaneous expression of three or more functions. These pie charts show average HIV-specific CD3+CD4-CD8+T-cell functions stratified by IL-2+ secreting T-cells. Three subjects: (i) UG006 (HLA B*5702, clades D, A), (ii) UG007 (HLA B*5702/03, clades D, B) and (iii) UG060 (HLA B*5704, clades A1, C) are indicated. Pie slices show proportions of CD8+ T-cells that concurrently express four (light blue), three (dark blue), two (light brown) and one function (dark brown). Pie arcs represent exclusive contributions of Perforin, TNF-α or IFN-γ to the total response within IL-2 secreting HIV-specific CD8+ T cells. For each subject, overall contribution of virus-specific Perforin T-cells is indicated above the corresponding pie chart. (For interpretation of the references to colour in this figure legend, the reader is referred to the web version of this article.)

In HLA B*57/*5801 subjects, measurable CD4+ T-cell responses (Fig. 2A) and CD8+ T-cell responses (Fig. 2B) were detected at varying frequencies against at least one KF11 variant. Assessment of the relationship between host HLA B alleles, possession of A163X, and the associated KF11-specific CD8+ T-cell response revealed no evidence for superior T-cell response(s) in clade A1- than clade D-infected subjects. Overall, frequencies of the detected virus-specific Perforin and CD8 + T-cell polyfunctionality were equivalent or higher in some clade D infected individuals (Fig. 3A and B, respectively) compared to A1 subjects (Fig. 3C).

**Fig. 4:**
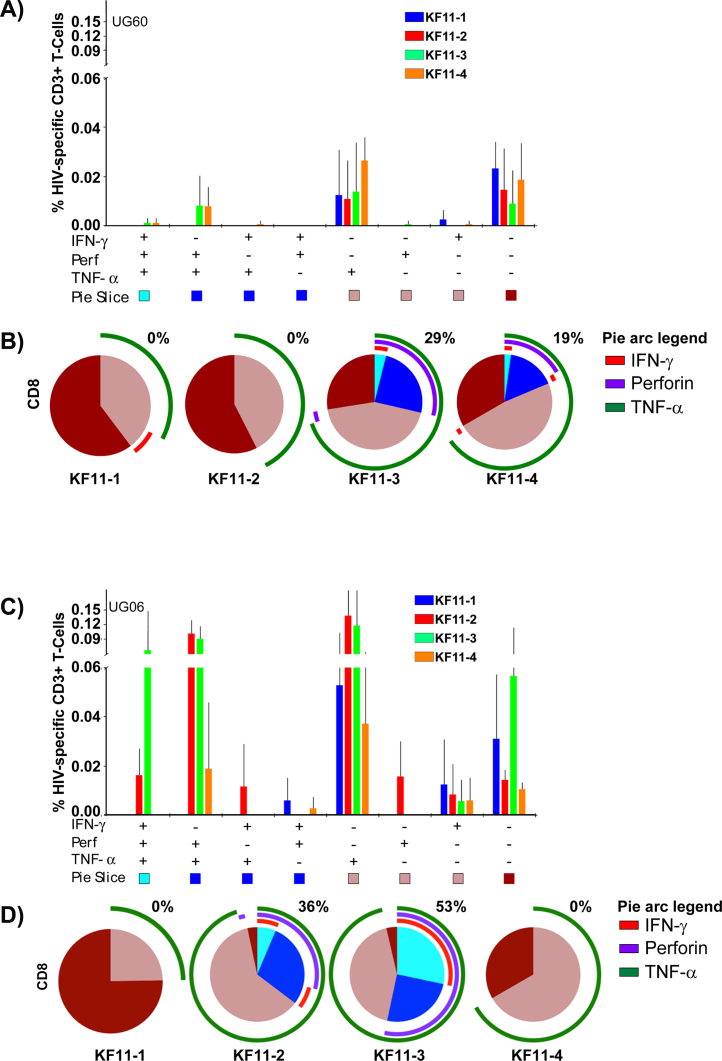
Polyfunctional T-cell responses to the KF11 epitope variants were detected at comparable frequencies across clades. This figure illustrates concurrent expression of IFN-γ, Perforin and TNF-α within virus-IL-2-secreting virus-specific CD8+ T-cells following stimulation with KF11-1 (blue bars), KF11-2 (red bars), KF11-3 (green bars) and KF11-4 peptides (orange bars). Distribution of 8 distinct functional subsets in one clade A1, B*5704 subject is illustrated (A). The *x*-axis represents positive (+) and negative (−) responses within each combination of IFN-γ, Perforin and TNF-α functional subset. The *y*-axis represents the percentage of IL-2-secreting virus-specific CD8+ T-cells contributing a given functional subset. Bars and error bars indicate means and standard deviations, respectively. Pie charts compare the average functionalities of IL-2 secreting; virus-specific CD8+ T-stratified according to the four KF11 variants (B). Polyfunctionality was defined as the concurrent expression of ≥3 functions. Pie slices denote proportions of CD8+ T-cells co-expressing 4 (light blue), 3 (dark blue), 2 (light brown) and 1 function (dark brown). Percentages of IL-2 secreting polyfunctional CD8+ T-cells are given above each pie. Pie arcs represent proportions of the CD8+ T-cell response containing Perforin (purple arcs), TNF-α (green arcs) and IFN-γ (red arcs). Likewise, relative contributions (C) and proportions (D) of virus specific CD8+ T-cells in IL-2 secreting subsets are illustrated in one clade D-infected, HLA B*5702 subject. (For interpretation of the references to colour in this figure legend, the reader is referred to the web version of this article.)

The CD8+ T-cell response to wild type KF11 (KF11-1) were of comparatively lower frequencies in both clade A infected (Fig. 4A and B) and clade D infected subjects (Fig. 4C and D). Secretion of TNF-α dominated the CD8+ T-cell response, while IFN-γ responses were marginal or absent. Wild type KF11 responses were largely monofunctional and lacked Perforin. On the other hand, variant responses were more polyfunctional and were detectable at comparable frequencies in both clades A1 and D subjects. Taken together, these data do not suggest any evidence for superiority of T-cell responses to KF11 in clade A1 infected patients compared to clade D infected patients.

### 3.7 Clade A sequences were subjected to greater selective pressure than clade D sequences

**Fig. 5:**
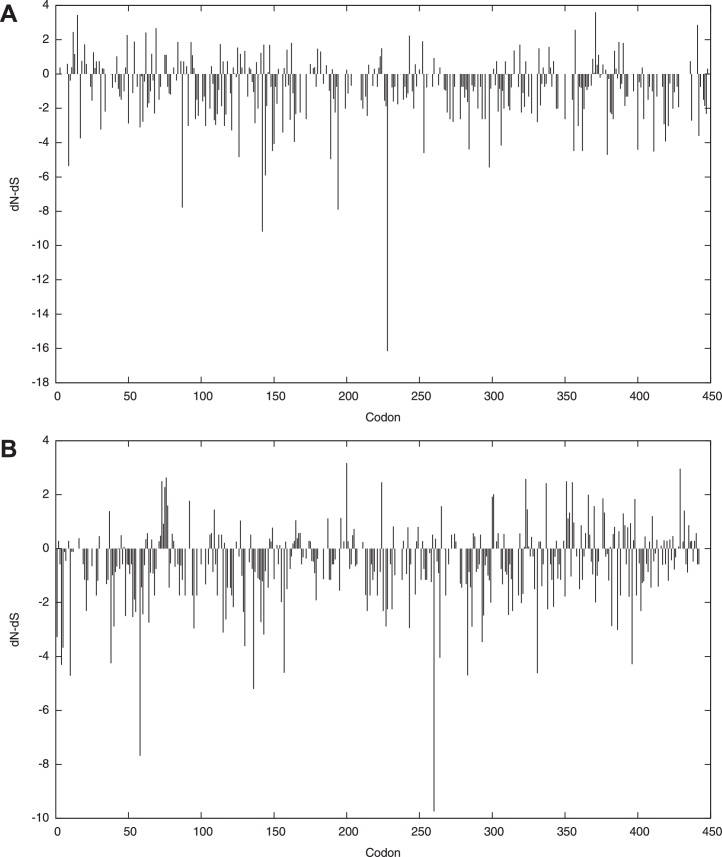
Rates of immune pressure across clades A and D sequences. This figure compares rates of non-synonymous (dN) and synonymous (dS) substitutions along the Gag protein codons, as a measure of immune selection pressure on clade A (A) and clade D (B) Gag protein sequences. Estimates of the phylogeny (topology and branch) and the codon-based substitution Bayesian model are shown. Total substitution rate and 95% CI for the posterior distribution estimated in the Bayesian Markov chain Monte Carlo (MCMC) analysis for clades A and D Gag sequences are illustrated.

We then evaluated the rates of non-synonymous (dN) and synonymous (dS) substitutions, as a measure of the selection pressure on the clades A and D Gag protein sequences, Fig. 5. The dN/dS ratio provides a measure of the selection pressure to the reference sequence. We found four positively selected sites, and 79 negatively selected sites in clade A sequences; while clade D had eight and 67 selected sites, respectively, 0.1 significance level. There was significantly more negative selection in clade A [mean dN/dS ratio 0.328214 (95% CI = [0.29962,0.358633]) than in clade D [mean 0.438708 (95% CI = [0.407466,0.471585]) sequences, continuous extension of the binomial distribution test, *p* = 5 × 10^−7^ (<0.005). We also observed an overall low synonymous substitution rate for HIV-1 clade A, which is known to be less pathogenic than HIV-1 clade D.

### 3.8 Binding affinity to the KF11 epitope was comparable across clades A and D

**Supplementary Fig. 2:**
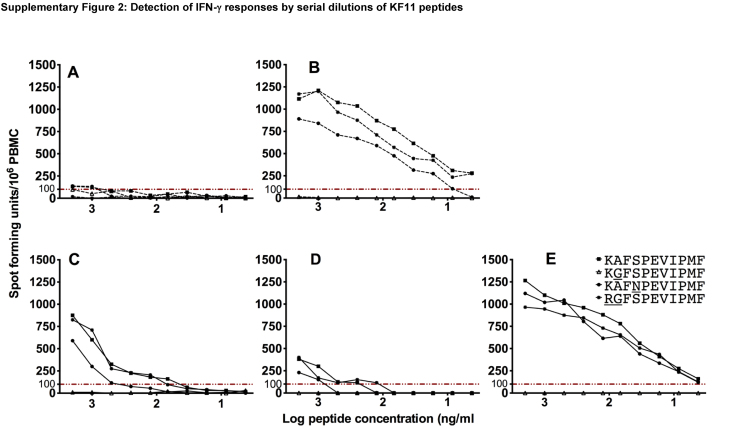
Detection of IFN-γ responses by serial dilutions of KF11 peptides. This figure illustrates KF11-specific IFN-γ ELISpot responses to varying concentrations of the four KF11 epitope variants varying from 2 μg/ml to 4 pg/ml. HLAB*57-restricted IFN-γ responses to clades A (dotted lines; A and B) and D peptides (solid lines; C–E) are shown.

**Supplementary Fig. 3:**
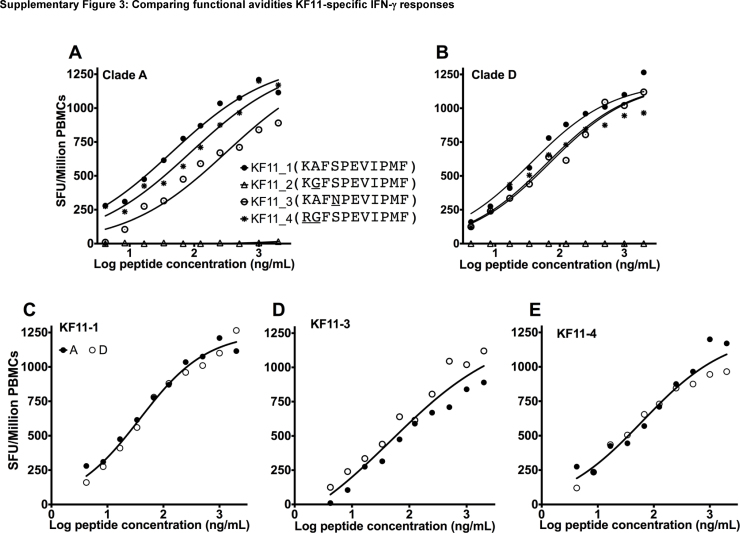
Functional avidities KF11-specific IFN-γ responses. This figure demonstrates dose–response sigmoidal curve fits comparing functional avidities of four HLA B*57-restricted KF11 epitope variants KAFSPEVIPMF (KF11-1), KGFSPEVIPMF (KF11-2), KAFNPEVIPMF (KF11-3), and RGFSPEVIPMF (KF11-4). Functional avidities were determined as peptide concentrations that yielded half the maximum number of spot forming units (SD50%). Functional avidities are compared acrossKF11 variants in clades A (A) and D subjects (B); and across clades for KF11-1 (C), KF11-2 (D) and KF11-3 peptide variants (E).

We finally evaluated whether clades A- and D-infected, HLA B*57 subjects differed in KF11 functional avidity. We detected varying levels of IFN-γ responses to KAFSPEVIPMF (KF11-1), KAFNPEVIPMF (KF11-3) and RGFSPEVIPMF (KF11-4) peptides in clade A (Supplementary Fig. 2A and B) and D (Supplementary Fig. 2C–E). Some low-grade ELISpot titres were observed in both clades. Wild type KF11 responses were of higher avidity than mutant responses, Supplementary Fig. 3A and D, clades A and D respectively. Sigmoid curves from two datasets that showed more robust IFN-γ responses (Fig. 2B and E) revealed no difference in functional avidities across clades A and D, Supplementary Fig. 3.

Supplementary material related to this article can be found, in the online version, at http://dx.doi.org/10.1016/j.vaccine.2015.02.037.

## 4 Discussion

The greatest impact on HIV disease may be mediated by HLA B-restricted induction of effective virus-specific CD8+ T-cell responses to certain Gag T-cell epitopes [18]. The resultant protection is achieved through selective pressure [10,11,18,21] on the structurally constrained ISW9, KF11 and TW10 Gag p24 CD8+ T-cell epitopes, generating variants with impaired replicative ability [11,12,34,35]. Ugandan is concurrently infected with nearly equal proportions of co-circulating clades A and D viruses [36]. We evaluated whether these virus variants that confer apparent advantage to hosts, accumulate at similar frequencies across clades A and D infection; and determined how frequencies of those variants correlate with the concomitant T-cell responses. We combined measurements of adaptive T-cell functionality with ISW9, KF11 and TW10 Gag p24 epitope sequence data to demonstrate that the A163X mutation in KF11 was more associated with clade A1-infection; the compensatory mutation I147X, known to restore immune recognition of the ISW9 epitope [37], was more frequent in clade A1 subjects; but frequencies of TW10 epitope polymorphisms did not significantly vary across clades.

Accumulation of HLA-restricted mutations in HLA matched hosts has been demonstrated before [38,39]. In matched hosts, HLA-B*57-restricted A163X escape mutation in the KF11 epitope has been shown to result in reduced plasma viral loads [11,40]; while I147X compensatory mutation has been linked to restoration of ISW9 epitope recognition [37]. Structurally constrained Gag p24 T-cell epitopes would be expected to yield similar escape pathways in allele presenting subjects. However, only a third of HLA matched hosts mount an epitope-specific response [41]; and the six major MHC determinants of HIV-1 protection in Caucasians [42] lacked protective associations in African cohorts [26]. It is also unclear why the widely protective HLA B*5801 [18,43,44] lacked protection in clade A-infected Rwandans [45]. Others also demonstrated higher A163X frequencies among clade A subjects [27]. However, it remained unclear whether this outcome depended on differences in concurrent adaptive responses. The higher rates of A163X mutation observed in HLA matched and mismatched clade A1 subjects in our population suggest better tolerance of those changes by clade A viruses. Differential clade-based outcome was also reported in clades B and C infected populations; where it was linked to functional constraints imposed by HIV clade on specific Gag residues [46]. Here, we found evidence for greater selective pressure on clade A Gag compared to clade D Gag; implying that the clade-specific outcome were linked to differences in immune pressure on the infecting virus clades. These findings are in line with those recently reported, which demonstrated clade-associated superior targeting of Gag among clade C- compared to clade B-infected, HLA B*0702 subjects [47].

Indisputably, T-cells exert strong selection pressure on HIV-1, generating HLA allele-restricted mutants that evade subsequent T-cell responses [48,49]. Emergence of HLA-B*5701-restricted variants has been linked to superior maintenance of IL-2 and Perforin, and persistence of polyfunctional CD8+ T-cell responses [50]. Superior virological control has been linked to greater functional avidity of the T-cell responses [31]. Here, we found no evidence for lower KF11 binding affinities to clade D peptides. However, avidity comparisons reported here needs to be interpreted with caution, as there were limitations of sample size. A larger sample size will be required to adequately evaluate relationships with functional avidity. In this study, plasma viral loads were eight-fold lower in presenting hosts bearing the A163X mutation suggesting the likelihood for potential T-cell involvement. The evaluated epitope-specific IFN-γ and polyfunctional responses were similar across clade A1 and D suggesting that clade-specific variations observed were not merely due to differences in the adaptive T-cell responses assessed here. The detected preservation of wild-type KF11 despite existence of epitope-specific polyfunctional CD8+ T-cell responses suggests that either the responses to clade D infection was ineffective or the virus was less tolerant of the mutations. We observed that responses to mutant epitopes were polyfunctional, while CD8+ T-cell polyfunctionality to the wild type epitopes had diminished. Possibly the responses were exhausted; specimen limitations did not allow for evaluation of exhaustion status of the antigen-specific T-cells. It is not surprising that we detected few TW10 directed epitope escape. The evaluated subjects were all chronically infected; yet TW10 epitope is targeted by acute CD8+ T-cell responses while the B*57-restricted KF11 epitope is dominant in chronic infection [18].

Overall, association of clade A1 subjects with escape mutations that are known to confer protection supports the notion that clade A infection with slower disease progression than D [1–3,10]. Overall, the data also support others that linked Gag targeting with improved outcome [10]. While the associations we report here are significant, there were some limitations. First of all, a longitudinal follow up would have better enabled evaluations of relationships between the various factors and the trends in evolutionary pathways. Secondly, these subjects were randomly selected for HLA B*57/5801 expressions without bias; finding of such associations in relatively few subjects suggests prevailing circulation of KF11 polymorphisms in clade A1-infected populations. However, a larger cohort will possibly allow for evaluation of correlations with rarer determinants. Lastly, further studies will be necessary to evaluate how these persistent dominant antigenic stimulations translate into T-cell exhaustion.

Overall, these data imply that the existing obstacles to HIV T-cell based vaccines may be further complicated by clade-associated differences in selective pressure on known HLA associated Gag outcomes. Even if critical epitopes remained as targets, their continued accumulation and adaptation in infected populations may lower the cost to viral fitness possibly reducing the host benefit. If the wild epitopes remain preserved, T-cell responses will likely persist but may become exhausted and functionally impaired due to continued antigenic stimulation. The data also raises hope that increased selective pressure on Gag will contribute to protection irrespective of host HLA alleles. The work presented here highlights the need for improved understanding of how HIV-1 diversity influences population-specific correlates of protection. The data have implications for T-cell vaccine approaches targeting highly conserved virus sequences to attenuate the infecting virus, and underscore the need to understand the direction of virus evolution when designing HIV T-cell vaccines.

## Conflict of interest

There are no financial, consultant, institutional and other relationships that might lead to bias or a conflict of interest.

## Author contributions

All authors made substantial contributions as follows: (i) the conception and design of the study (J.S., P.K., R.N.), or acquisition of data (R.N., S.M., B.M.) or analysis and interpretation of data (J.S., R.N., S.M., N.N.), (ii) drafting the article or revising it critically for important intellectual content (J.S., R.N., N.N., F.G., P.K.), (3) final approval of the version to be submitted (J.S., F.G., P.K.).

## Figures and Tables

**Table 1:**
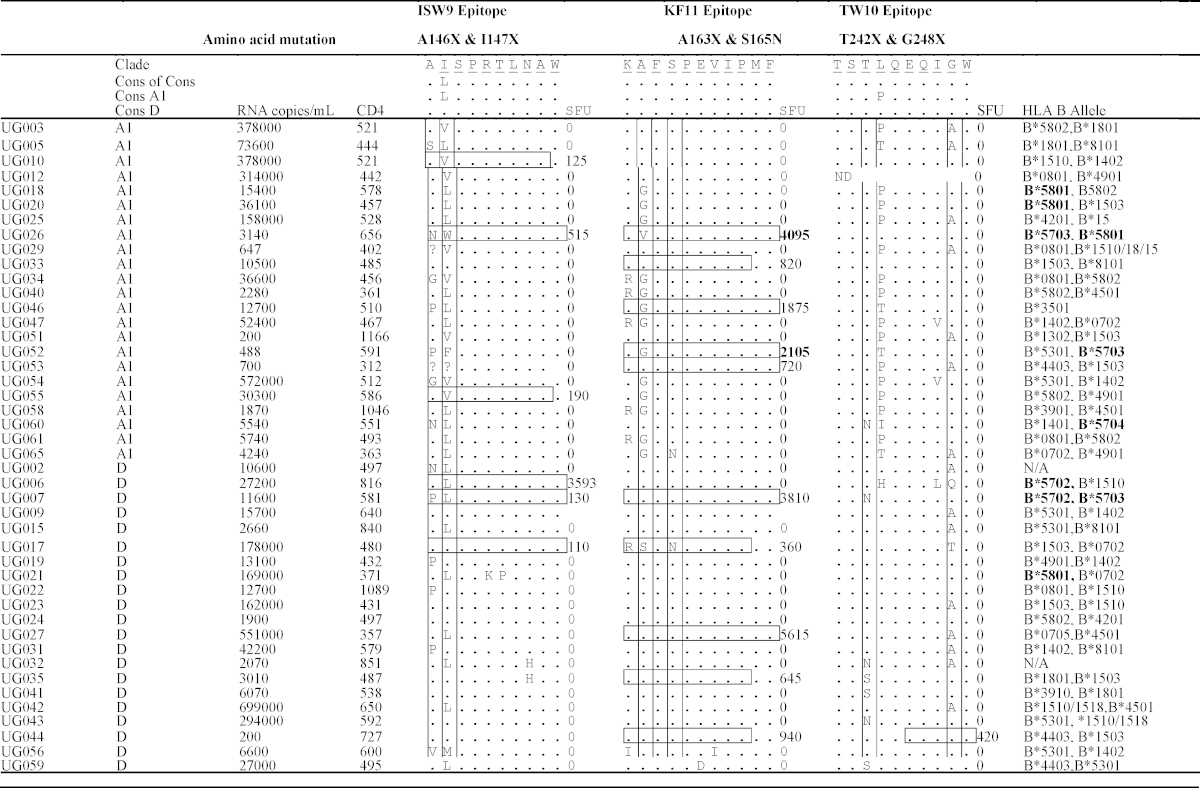
Characteristics of the study populations and their infecting viruses. This table illustrates characteristics of the study participants. Sequence variations in key Gag epitopes KAFSPEVIPMF (KF11) residues 162–172; ISPRTLNAW (ISW9), residues 147–155 and TSTLQEQIGW (TW10) residues 240–249 are compared. Horizontal boxes highlight peptides that elicited IFN-γ response in that patient. Spot Forming Units per million PBMCs are indicated for cases where interferon (IFN)-γ responses were detected. HLA alleles known to present KF11, ISW9 and TW10 epitopes are highlighted in bold. Induced Spot forming units are highlighted in bold for two cases where IFN-γ responses were maintained despite the presence of an A163X escape mutation.
